# Modeling habitat suitability and connectivity for the sole endemic genus of Italian vertebrate: present and future perspectives

**DOI:** 10.1186/s12983-025-00562-6

**Published:** 2025-05-26

**Authors:** Davide Serva, Ilaria Bernabò, Viviana Cittadino, Antonio Romano, Francesco Cerasoli, Maurizio Biondi, Mattia Iannella

**Affiliations:** 1 Department of Life, Health & Environmental Sciences - LACEMOD Lab, University of L’Aquila, Polo Coppito 1, Via Vetoio 40, 67100 L’Aquila, Italy; 2 Department of Biology, Ecology and Earth Science, University of Calabria, Via P. Bucci 4/B, 87036 Rende, Italy; 3 Consiglio Nazionale delle Ricerche, Istituto per la BioEconomia, Via dei Taurini, 19, 00185 Rome, Italy

**Keywords:** Climate change, Ecological corridors, Endemism; Habitat connectivity, Hybridization, *Salamandrina*; Spectacled salamanders, Species distribution models

## Abstract

**Background:**

Amphibians are the most globally threatened vertebrates, facing a particularly high risk of extinction in some regions, such as the Mediterranean basin. Within this region, the genus *Salamandrina*, comprising two species of notable conservation concerns, managed to persist throughout historical climate changes exclusively along the Italian peninsula. Among the main threats to this genus are habitat loss and climate change, as this salamander is adapted to humid forests and relies heavily on water sources, such as small streams, for reproduction. In this study, we employed fine-scale species distribution models (SDMs) to estimate areas projected to remain suitable for *Salamandrina* species in the future and areas expected to become unsuitable, incorporating bioclimatic, topographic, and habitat variables. We also evaluated landscape connectivity and identified ecological corridors that could facilitate movement through circuit-theory techniques, analyzing potential changes under different scenarios. Additionally, considering known hybridization events in a contact zone between the two species, we measured connectivity to assess whether this process might change in the future.

**Results:**

We found several suitable areas that mostly align with the known ranges of both species. Future projections showed an overall decline in habitat suitability, with a northwestern shift. While strong in certain areas, landscape connectivity is expected to decrease overall. Specifically, our results revealed several corridors for *S. perspicillata* (the northern species), with higher connectivity values in the Central Apennines. For *S. terdigitata* (the southern species), a crucial corridor in the Catena Costiera Massifs, in the western southernmost region of the Italian peninsula, connects two key conservation areas. In the contact zone, we identified corridors that could favor hybridization between the species, with predictions suggesting an increase.

**Conclusions:**

Our findings inform the long-term conservation of a unique salamander genus. Strengthening conservation measures on *Salamandrina* species in areas predicted to become unsuitable or in areas that could become suitable and serve as ecological corridors will be critical. Furthermore, future monitoring efforts should focus on the corridors identified in the contact zone to assess whether the hybridization process is ongoing and at what rate.

**Supplementary Information:**

The online version contains supplementary material available at 10.1186/s12983-025-00562-6.

## Background

The awareness of amphibians' negative conservation status, as the most endangered vertebrate class, dates back over 30 years [1–4]. Today, 43% of amphibian species globally face extinction, with regions like the Mediterranean basin, especially the Italian peninsula, experiencing significant declines [4, 5].

The genus *Salamandrina* Fitzinger, 1826, an ancient lineage of the family Salamandridae, is endemic to the Italian peninsula and includes two species—*Salamandrina perspicillata* (Savi, 1821) and *Salamandrina terdigitata* (Bonnaterre, 1789). Known as spectacled salamanders, these species are distinguished by unique morphology [6] and have been extensively studied for their evolutionary and conservation significance [7–9]. Fossil records [8] reveal a once broader historical distribution across Europe, now reduced to peninsular Italy, probably due to climate constraints over millions of years. In the past 120,000 years, the species' ranges have fluctuated, with distinct and sometimes opposing climatic needs [9]. Projections for future climatic conditions indicate a decline in bioclimatic suitability for the genus *Salamandrina* under two future scenarios [8]. Genetically, the genus includes two recently separated species based on nuclear and mitochondrial data [10–12]. Morphometric analysis alone cannot reliably distinguish them [6]. Genetic studies estimate their divergence between 2.5 [13] and 11.5 million years ago [7, 11]. The species have distinct glacial refugia: *S. perspicillata* in southern Latium (central Italy) and *S. terdigitata* in Calabria (southern Italy), with multiple refugia for the latter. Despite demographic differences [13], both species meet in a secondary contact zone, the Volturno Valley in Campania, where hybridization occurs [7, 14, 15]. However, no spatial predictions exist for the future distribution of these species. As the oldest lineage within Salamandridae, *S. perspicillata* was recently reclassified from “Least Concern” to “Endangered” due to disease risks [16], though this assesment is debated [17]. These species are protected by the EU Habitats Directive (92/43/EEC) and other laws, with Protected Areas (PAs) playing a key role in their conservation.

Given the effects of climate and land-use change, it is crucial to predict potential shifts in habitat suitability and identify areas where conditions may improve or deteriorate. Conservation efforts should target these areas, factoring in bioclimatic and finer-scale predictors. Additionally, maintaining landscape connectivity is essential, particularly for species with limited movement, like the spectacled salamanders. Studies suggest their movements are typically short (< 1 km), with high genetic differentiation even over small distances [13].

This study addresses critical knowledge gaps about the future habitat suitability of both *Salamandrina* species under climate change. Using a framework that includes climatic, land-use, and topographic predictors, we first create Ecological Niche Models (ENMs) and refine them into Species Distribution Models (SDMs) [sensu 18]. These models assess landscape connectivity across the species ranges. Additionally, we evaluate how changing climate conditions might influence hybridization in the sympatric Volturno Valley, where hybridization events have been observed. By focusing on this area, we aim to determine if climate change could facilitate introgression by connecting currently suitable areas for both species. This approach will inform conservation strategies, helping to identify areas where efforts should be concentrated to protect this endemic lineage.

In summary, our findings could guide strategic conservation planning, enhance the effectiveness of territorial protections, and support the long-term preservation of these unique salamander species.

## Methods

### Study area and target species

*Salamandrina perspicillata* (Savi, 1821), the Northern spectacled salamander, and *Salamandrina terdigitata* (Bonnaterre, 1789), the Southern spectacled salamander, are associated with similar environmental conditions, preferring moist forest habitats. The life cycle is mainly terrestrial, with females exhibiting an aquatic phase during spawning, selecting slow-moving waters, such as small streams, springs, and drinking-troughs [19].

The distribution of the genus *Salamandrina* spans across the Apennine chain, with the range of *S. perspicillata* from the Piedmont to the Volturno Valley in Campania, while *S. terdigitata* occurs from the Volturno Valley to the northern Apulia and Calabria [6, 20]. Despite their parapatric distribution, there is a contact zone in the Volturno Valley, occurring within a larger hybrid zone hosting syntopic localities [7, 13–15].

We gathered occurrences from the authors’ field observations and published literature for both species, spatially filtering, for the latter, records with GPS precision (~ 100 m precision of the locality); we obtained a distribution consistent with the current known ranges [16] of both species (Additional file 1, Fig. S1). To account for possible spatial biases among occurrence localities, the presence points for both species were filtered to obtain a comparable spatial resolution between occurrences and predictors, as recommended in [21]. The final dataset contains: 298 occurrences for *S. perspicillata* and 128 for *S. terdigitata*.

To model habitat suitability (see next) across a spatially realistic extent, we defined the calibration area as the Italian peninsula, encompassing all of its biogeographic sectors, in accordance with the BAM framework [18]. This region, which slightly exceeds the species' current distribution, represents a biogeographically and ecologically coherent area realistically accessible to the genus *Salamandrina* over historical times. This choice allowed us to calibrate the models while avoiding underestimation of the species’ fundamental niche.

### Environmental variables

Considering the ecology of both species, we used bioclimatic, topographic, and habitat-related variables in our analysis (Additional file 1, Table S1).

For the climatic facet, we downloaded nineteen bioclimatic variables from WorldClim (version 2.1, 30 arc-seconds spatial resolution, ~ 1 km) [22] for both present and future conditions (2030, 2050, and 2070). For each future scenario, we selected the Shared Socioeconomic Pathways (SSPs) 1.26, 2.45, 3.70, and 5.85 to involve all possible trajectories [23]. To reduce the variability potentially introduced by using individual General Circulation Models (GCMs) in future projections [24], we selected three different GCMs: BCC-CSM2-MR [25], IPSL-CM6 A-LR [26], and MIROC6 [27].

Topography was assessed by including Slope and Aspect as predictors. Starting from a Digital Elevation Model (EU-DEM v.1.1) with a precision of ~ 25 m, downloaded from (https://land.copernicus.eu/en), we calculated both topographic predictors mentioned above, using the ‘Surface Parameters’ tool in ArcGIS Pro [28].

To account for species’ habitat preferences, we downloaded the EUNIS habitat categories from the European Environment Agency (https://www.eea.europa.eu/data-and-maps/data/ecosystem-types-of-europe) at a resolution of ~ 100 m. Given the species’ reliance on small rivers for reproduction and larval development, we downloaded the river data from HydroRIVERS (https://www.hydrosheds.org/products/hydrorivers). We then calculated the Euclidean distance after selecting small tributaries and streams by excluding those with high Strahler order (> 3), as not used by the species [29]. Thus, we finally obtained a distance raster, in which each cell represents a distance from the selected streams. Moreover, considering the importance of small water bodies for both species, we compiled a database of pools, springs and drinking-troughs, which could potentially be used during the breeding season. We then calculated the Euclidean distance among these water bodies similarly as performed above. We used the original resolution from WorldClim for the ENMs, then resampling the spatial predictions to a resolution of 100 m, to obtain a consistent spatial resolution of 100 × 100 m for the fine-scale modeling.

### Environmental niche modelling

We accounted for multicollinearity among the nineteen WorldClim bioclimatic variables (the ones used to obtain the ENMs) by computing the Variance Inflation Factor (VIF) through the ‘vifstep’ algorithm of the ‘usdm’ R package [30], excluding those variables with VIF ≥ 10 [31].

We then used the retained variables at their native resolution to model climatic suitability for *S. persipicillata* and *S. terdigitata* across the study area taking advantage of the ‘biomod2’ R package [32], which allows to combine single ENMs, fitted through different algorithms and/or on different presence-(pseudo)absence datasets, into ensemble models. To fit the ENMs, we chose three algorithms, representative of a range of techniques spanning from traditional multivariate regression (Generalized Linear Models, GLM) to ensembles of classification/regression trees (Random Forest, RF) and hybrid regression-boosting approaches (Gradient Boosting Models, GBM).

Prior to model fitting, we used the ‘BIOMOD_FormatingData’ function to generate, for each species, 10 sets of pseudo-absences, each containing 1000 points randomly drawn within those areas showing climatic conditions outside the 95th percentile of the rectilinear surface range envelope (SRE) estimated from presence points (‘PA.strategy’ = ‘sre’, ‘sre.quant’ = 0.025) [33]. Each set of pseudoabsences was then collated to the thinned presence points, obtaining 10 presence-pseudoabsence datasets (hereinafter, ‘Pres-PseudoAbs’) for each species.

Then, to lower the risk of obtaining biased estimates of model predictive performance due to spatial autocorrelation in climatic conditions, we estimated, for both species, the spatial autocorrelation range (SAR) and later implemented checkerboard cross-validation (CV) [34]. To estimate the SAR, we: (i) fitted a preliminary ENM for each species × algorithm × “Pres-PseudoAbs” combination; (ii) computed the residuals of model predictions on the training data; (iii) used the “ncf” R package [35] to calculate Moran index (Moran’s I, a metric of spatial autocorrelation) on such residuals at increasing inter-point distances, later deriving a correlogram which permits to visualize how Moran’s I varies with inter-point distance. The distance at which Moran’s I from model residuals stably approaches 0 is generally interpreted as a proxy for SAR [34]. Then, we took advantage of the “blockCV” package to create the checkerboard CV folds, with block size higher than the estimated SAR [34, 36]. Consequently, we adopted a checkerboard block size corresponding to the spatial scale at which residual spatial autocorrelation was no longer detected. Regardless of whether this distance is considered large or small in absolute terms, it is the one that ensures spatial independence between training and test sets, thus substantially reducing prediction bias caused by autocorrelated data. With this approach, we followed the recommendations by Roberts et al. [34], who emphasize that block size should be informed by the scale of spatial autocorrelation to ensure unbiased model evaluation.

Both the preliminary ENMs and those fitted within the BIOMOD pipeline (see below) were parameterized as follows: for GLM, we fitted additive models, using quadratic polynomials for predictors and binomial error distribution with logit link function for the response variable (i.e., presence-absence); for GBM, we fitted 5000 trees, setting three-way interactions for the predictors and bernoulli distribution for the response variable, with minimum number of observations in the terminal nodes = 10, shrinkage parameter = 0.001, ‘bag.fraction’ = 0.5, three randomly generated CV folds for internal validation with training fraction = 0.8; for RF, we fitted 500 classification trees, with two predictors randomly chosen at each branch split (i.e., ‘mtry’ = 2) and minimum size of terminal nodes set to 5 observations.

The ‘BIOMOD_Modeling’ function was then used to fit the ENMs for each Pres-PseudoAbs dataset, assigning an overall equal weight to presences and pseudoabsences; this weighting scheme usually results in better discrimination performance when the generated pseudoabsences are notably more than the available presences [37]. The predictive performance of the single ENMs on the test data selected through checkerboard CV (CV.strategy = ‘user.defined’) was quantified considering as validation metrics the Boyce index and the True Skill Statistic (TSS), the former being particularly suitable when absence data from structured surveys are not available [38]. The ENMs attaining relatively high predictive performance on test data (i.e., Boyce index > 0.7 and TSS > 0.5) were selected as input for the ‘BIOMOD_EnsembleModeling’ function to generate an ensemble model (EM), where the contributions of the single ENMs were weighted based on the corresponding Boyce index (metric.eval = c(‘BOYCE’), em.algo ='EMwmean'). Finally, we projected the EM across the study area for both current conditions and each future scenario through the ‘BIOMOD_EnsembleForecasting’ function.

For each of the 24 future scenarios (i.e., 3 GCMs × 3 projection years × 4 SSPs), we also quantified the degree of extrapolation (i.e., discrepancy between the calibration and the projection conditions) by computing the Multivariate Environmental Similarity Surface (MESS) [39] through the ‘mess’ function of the ‘dismo’ package [40]. The MESS outputs were then included in the Multivariate Environmental Dissimilarity Index (MEDI) algorithm [41] to derive, for each year × SSP scenario, a weighted average of the model projections obtained from the three different GCMs.

### Fine-scale modeling

To deepen the knowledge about species’ distribution we used the ‘Suitability Modeler’ widget in ArcGIS Pro. This widget provides an interactive framework that allows the user to transform the values of each predictor to a common suitability scale, assign a weight to each variable, and combine them to create the final suitability map, in a process which was shown to increase predictive performances [42]. In this framework, each predictor is reclassified based on the ecological preferences of the target species, transforming the data using the function that best approximates the relationship between species and environmental predictors.

Information about habitat, slope, aspect, and distance to water bodies was extracted in occurrence localities for both species through the ‘Extract multi values to points’ tool in ArcGIS. Relative frequencies were then calculated for each predictor/occurrence locality, and a curve was fitted to detect the best function that could approximate the species’ habitat preferences.

Next, each variable was transformed, and a specific weight was assigned to each variable, based on both literature [29, 43, 44] and on an expert-based opinion. For future fine-scale predictions, we incorporated climate-change scenarios by updating the bioclimatic variables while maintaining the other predictors fixed, as future projections for habitat-related variables at a high spatial resolution are currently lacking. As such, we applied the same transformations and variable weights used for current conditions to ensure consistency across scenarios. Finally, all the reclassified environmental variables and the outputs from the Ecological Niche Modelling were merged to build the final ‘weighted’ species distribution model for each species and each scenario considered. Details about the specific transformations applied to each environmental predictor and the relative weight are available in Additional file 1, Table S2.

The predictive performance of the weighted SDMs, obtained for current conditions, was evaluated by computing the continuous Boyce index (B), which ranges from − 1 (counter prediction) to + 1 (optimal prediction) [45, 46] through the'ecospat’ R package [47] based on occurrence localities of each target species.

### Current and future connectivity assessments

To assess the functional connectivity of both the target species, we utilized Circuitscape, implemented in Julia language (v1.9.2) [48, 49]. Circuitscape is the reference software for circuit-theory techniques and is based on the random-walk assumption, meaning that individuals moving across a landscape have no knowledge of surrounding habitat patches [48, 50]. Since Circuitscape requires a resistance (or conductance) surface as input, along with focal nodes (i.e., points or polygons between current will be computed), we converted the SDMs obtained for current and future scenarios into resistance layers. Thus, we first normalized the SDMs to a 0–1 scale. Then, we added important barriers to the SDMs, such as roads and urban areas. Information for roads was obtained from OpenStreetMap (https://www.openstreetmap.org/), selecting only major roads (i.e., in our case, highways, trunk, primary and secondary roads), while Urban areas were extracted from the Corine Land Cover map (https://land.copernicus.eu/pan-european/corine-land-cover/clc2018). Those barriers were added to the SDMs, assigning to them the highest resistance value (i.e., the lower suitability value) with the ‘Mosaic to new raster’ tool, which merges multiple raster datasets into a new one. Finally, a negative exponential function was applied to convert the SDMs into resistance layers, following the method outlined by Keeley et al. (2016) [51], with the constant *c* factor set to 4. These resistance layers, along with the occurrences of the target species (used as nodes), were used as input in Circuitscape, selecting the ‘pairwise’ mode. In this mode, current is computed between each pair of nodes, where each couple term is iteratively treated as both the source and the destination node. The resulting current map for each scenario represents the expected net probability of an organism (*Salamandrina* spp.) moving from one node to another [48]. Furthermore, to better assess how this connectivity might change in future scenarios, we calculated the Standardized Connectivity Change Index (SCCI) [42, 51]. This index yields values ranging from a gain (+ 1) to a loss (−1) of connectivity, with SCCI = 0 representing corridor maintenance.

### Hybridization chance assessment

As changes in connectivity induced by the ongoing global change may encourage movements of individuals to patches that have not been occupied in the species’ recent biogeographic history, we evaluated the possibility of this scenario occurring between the two species. First, we identified the sympatric area, where introgression had already been detected [7, 14, 15], by intersecting the ranges of both species, as provided by the IUCN Red List (https://www.iucnredlist.org/species/59468/89706354; https://www.iucnredlist.org/species/136135/89706481). We then created a 50 km buffer around the centroid of the sympatry area, to be sure to include the contact area entirely, using the ‘Buffer’ tool available in ArcGIS Pro. We assessed the ‘hybridization probability’ by calculating the potential future range shifts, predicted by our future habitat suitability predictions, and computing habitat connectivity using Circuitscape in ‘pairwise’ mode. For this latter, we generated a unique resistance layer by averaging the SDMs for the two species within the sympatric area. Specifically, a map pixel that is highly suitable for one species (e.g., a value of 1) and equally suitable for the other species would yield a high average value. Conversely, if a pixel is completely unsuitable for one of the species (e.g., a value of 0), the average value would be 0.5. We then added barriers and transformed the resulting layers into resistance surfaces using a negative exponential function, maintaining a constant *c* factor of 4, as previously reported. To evaluate the probability of contact between the two species, we calculated habitat connectivity by using all occurrences within the sympatric areas without distinguishing between the occurrences of the two species.

## Results

The preliminary ENMs showed Moran's I > 0 (i.e., spatial autocorrelation) at distances up to ~ 200 km, while autocorrelation tended to 0 at larger distances, with GBM showing wider variability than GLM and RF (Additional file 1, Fig. S2). Thus, 400 km was chosen as block size to structure the checkerboard CV folds (Additional file 1, Fig. S3). The ENMs fitted in BIOMOD and selected for ensemble modelling attained relatively high predictive performance on the spatially independent test data (*S. perspicillata*: Boyce index = 0.867 ± 0.082 (mean ± sd); *S. terdigitata:* Boyce index = 0.840 ± 0.089). The predictive performance of the weighted SDMs obtained after the fine-scale modelling phase was even higher (B_Wperspicillata_ = 0.913; B_Wterdigitata_ = 0.886). Predictions of climatic suitability from the ensemble models showed some areas with medium suitability (0.4 < suitability < 0.6) southwards of the current range for *S. perspicillata*, while climatically suitable conditions for *S. terdigitata* were mainly restricted to its current range, except for a narrow belt of suitable area along the central part of the Tyrrhenian coast and in northwestern Sardinia (where this species never occurred) (Additional file 1, Fig. S4).

The three most important bioclimatic variables within the Boyce-weighted EMs were: bio10 (i.e., mean temperature of warmest quarter) = 21.7%, bio01 (i.e., annual mean temperature) = 13.1%, and bio17 (i.e., precipitation of the driest quarter) = 12.3% for *S. perspicillata*; bio19 (i.e., precipitation of coldest quarter) = 25.5%, bio10 = 13.2%, and bio09 (i.e., mean temperature of the driest quarter) = 13.0% for *S. terdigitata.* The marginal response curves obtained from the same EMs showed contrasting patterns between the two species with respect to bio10 (i.e., with predicted suitability increasing with increasing in Bio10 for *S. perspicillata*, though the opposite for *S. terdigitata*), while higher precipitation was predicted to result in higher suitability for both (Additional file 1, Fig. S5).

The habitat selection assessed through the sampling procedure has shown for both species a preference for forest areas and areas near rivers, springs, and pools.

**Fig. 1:**
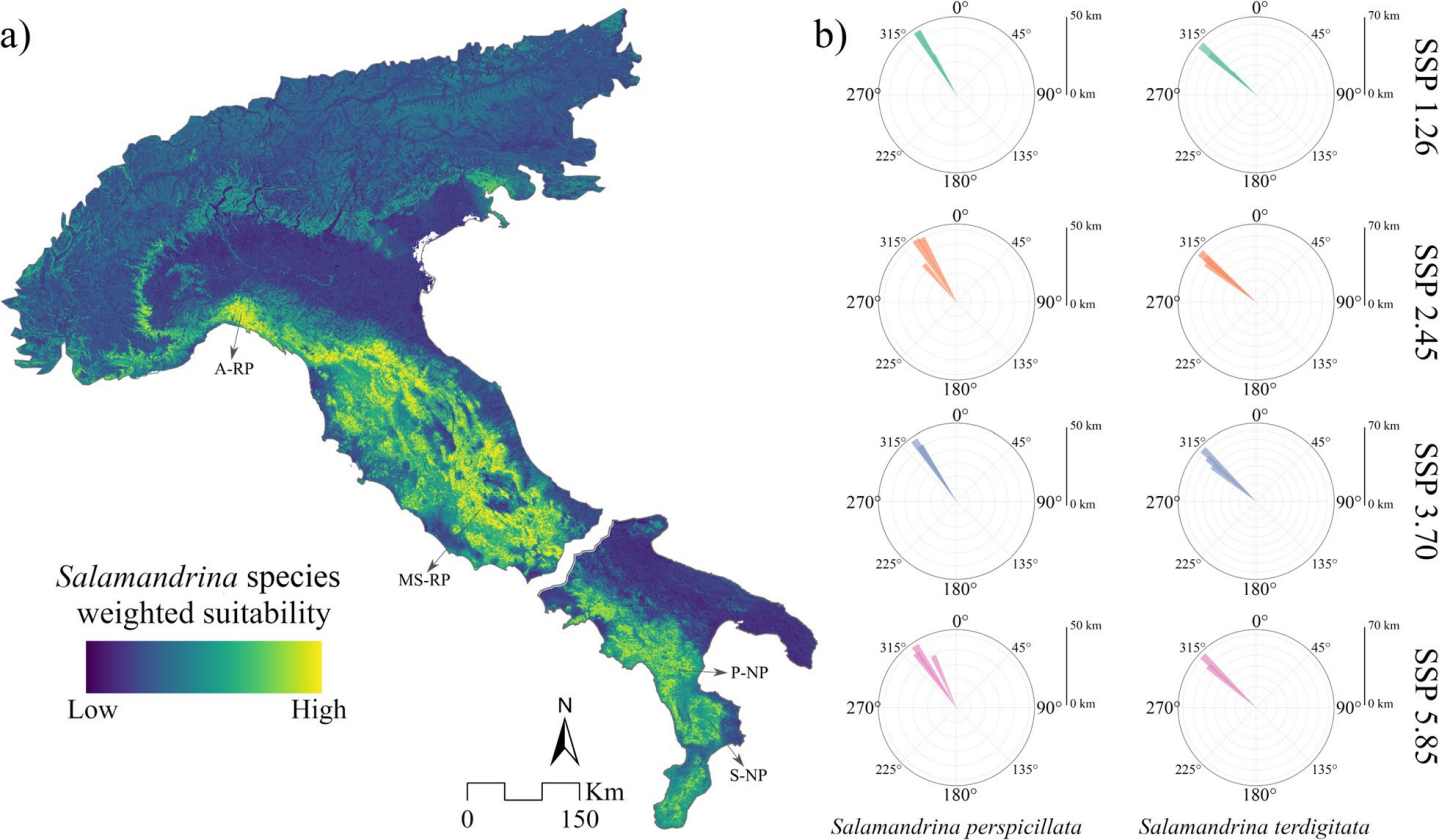
**a** Weighted habitat suitability after the fine-scale modeling approach for both species, and **b** predicted centroid shifts for both species in every scenario. Antola Regional Park (A-RP), Monti Simbruini Regional Park (MS-RP), Pollino (P-NP) and Sila National Parks (S-NP)

The weighted suitability for *S. perspicillata* showed several large suitable areas in the Central Apennines, between southeastern Latium and Abruzzi, such as close to the Monti Simbruini Regional Park (MS-RP) (Fig. 1a). There are also some large suitable patches in the Northern Apennines, extending up to the Antola Regional Park (A-RP) in Liguria region, where suitability rapidly decreases at the northern edge of its range (Fig. 1a). For *S. terdigitata,* suitable areas are primarily found from Campania to Calabria (Fig. 1a), with some particularly suitable small areas, such as the one close to the coast linking the Pollino (P-NP) and Sila National Parks (S-NP) (Fig. 1a). The centroid shifts showed a similar pattern across all scenarios for both species, with predicted range shifts moving northwestward (Fig. 1b). Longer distances were predicted for the 2050 and 2070 scenarios (Fig. 1b).

**Fig. 2:**
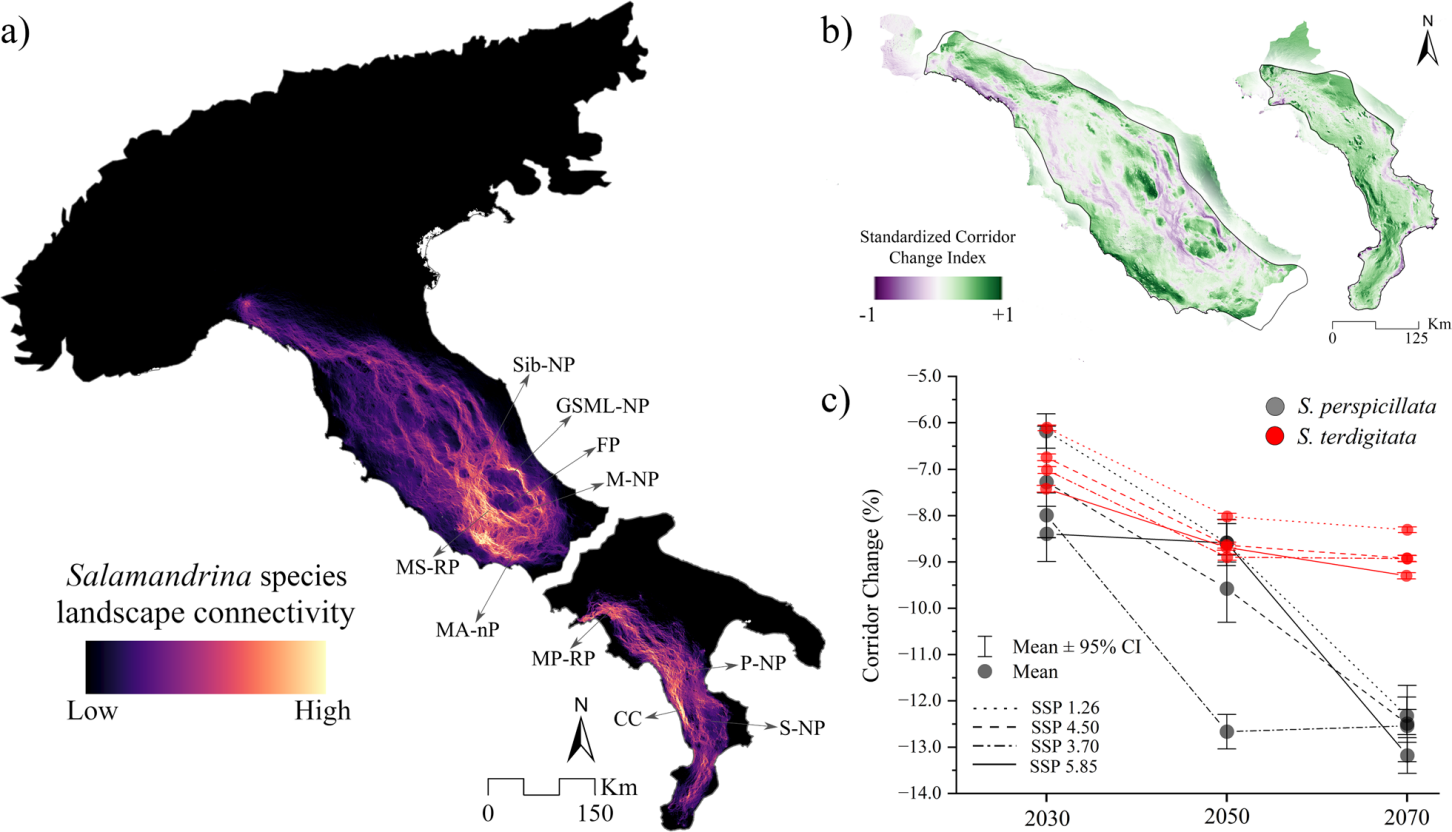
**a** Current landscape connectivity for *Salamandrina* species, **b** standardized corridor change index (SCCI) mean across all future scenarios, considering the area inside the ranges reported for both species from IUCN, and **c** predicted percent changes in connectivity across all scenarios for both species. Monti Simbruini Regional Park (MS-RP), Monti Aurunci natural Park (MA-nP), Fùcino Plan (FP), Gran Sasso and Monti della Laga National Park (GSML-NP), Majella National Park (M-NP), Sibillini National Park (Sib-NP), Monti Picentini Regional Park (MP-RP), Catena Costiera (CC), Pollino (P-NP) and Sila National Parks (S-NP)

Landscape connectivity predicted for current conditions for *S. perspicillata* showed a combination of diffuse connectivity, linear ecological corridors, and pinch-point areas (Fig. 2a). High diffuse connectivity is predicted in southern Latium (Central Italy), close to the Monti Simbruini Regional Park (MS-RP) and Monti Aurunci natural Park (MA-nP) (Fig. 2a). Furthermore, some corridors connect these areas with the Abruzzi region (Central Italy), encompassing two zones with low connectivity, approximately in the Fùcino Plan (FP) and Gran Sasso and Monti della Laga National Park (GSML-NP) (Fig. 2a). Through the Abruzzi region, corridors link the Maiella National Park (M-NP) to the Sibillini National Park (Sib-NP) towards the north (Fig. 2a). Further north, scattered corridors connect the central and northern Apennines up to the Liguria region (Fig. 2a). For *S. terdigitata*, key ecological corridors run through Campania, from areas close to Monti Picentini Regional Park (MP-RP) to Calabria (Southermost Italy), with increased connectivity predicted along the Catena Costiera (CC) between Pollino (P-NP) and Sila National Parks (S-NP) (Fig. 2a). Connectivity gradually decreases further south, especially in the coastal areas (Fig. 2a). The SCCI showed some differences between the species, with an overall increase in connectivity for *S. terdigitata* and a decrease for *S. perspicillata* (Fig. 2b). In particular, decreases in connectivity are predicted between Liguria and Tuscany, and across the main corridors linking Latium, Abruzzi, Umbria and Marche regions in the Central Apennines (Fig. 2b). In contrast, for *S. terdigitata*, an increase in connectivity is expected in the crucial corridor identified in the current scenario between Pollino (P-NP) and Sila National Parks (S-NP) (Fig. 2a and b). Further increases in connectivity are predicted southward, whereas a decrease is anticipated northward across Campania (Fig. 2b). When accounting for differences across future scenarios (Fig. 2c), higher decreases are predicted for *S. perspicillata*, with a medium reduction ranging from 7 to 13% (Additional File 1, Fig. S6). In contrast, *S. terdigitata* is expected to experience a minor decline, with an average decrease ranging from 6 to 9% (Fig. 2c; Additional File 1, Fig. S7).

**Fig. 3:**
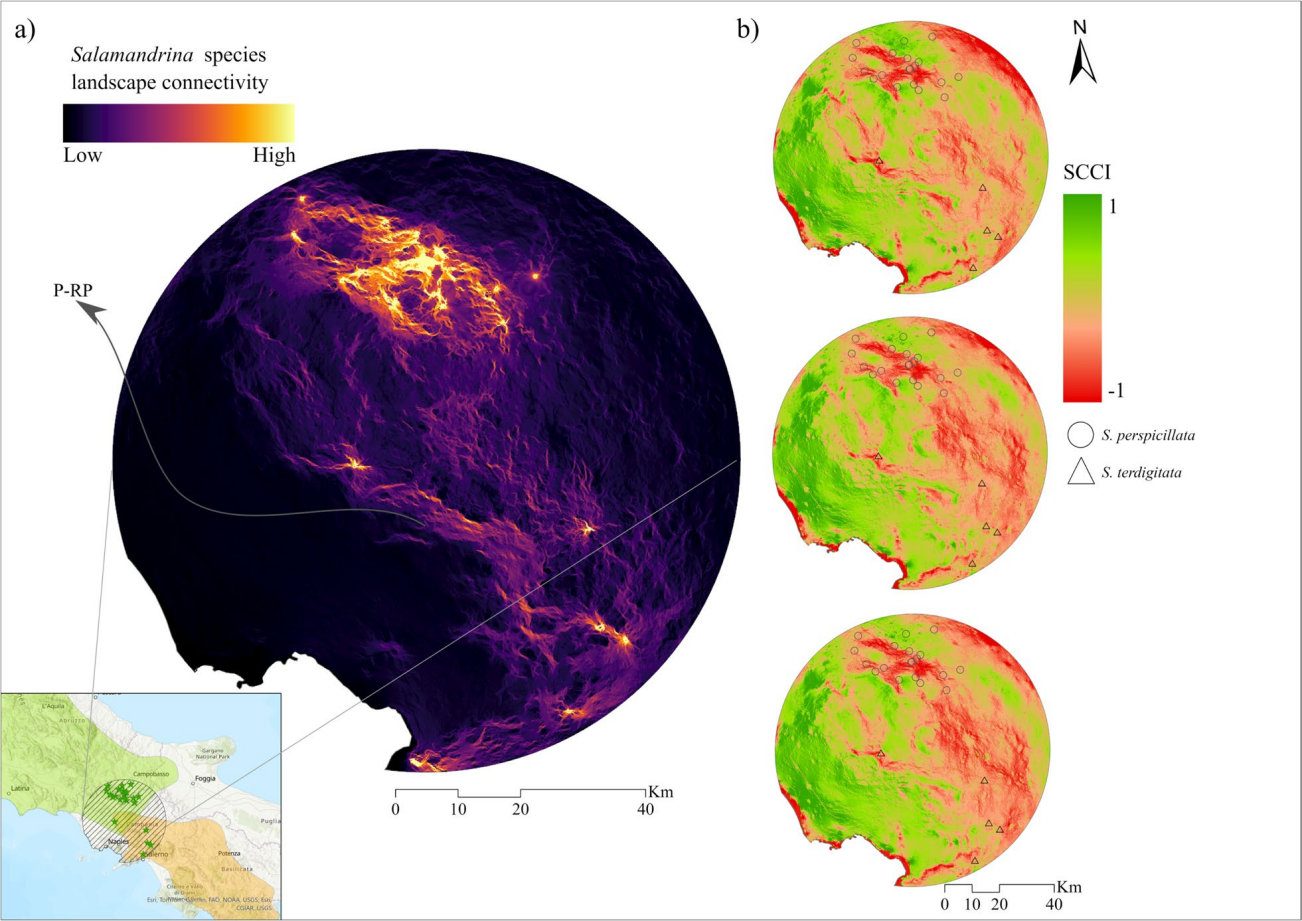
**a** Landscape connectivity within the introgression area for the current condition, and **b** standardized corridor change index (SCCI) for the future scenarios. Partenio Regional Park (P-RP)

Current landscape connectivity, as captured by Circuitscape in pairwise mode within the introgression area, showed higher connectivity values in the northern sector of the introgression area (Fig. 3a), where only *S. perspicillata* is reported by the IUCN species range. In addition, a corridor connects the central occurrences between the ranges of the two species, while some scattered corridors extend southwards (Fig. 3a). Despite the lower occurrences in the south, moderate connectivity is still observed. Overall, connectivity decreases dramatically in the more urbanized coastal areas outside the Apennine chain (Fig. 3a). In future scenarios, the SCCI showed an overall increase in connectivity in the west, where there are not many occurrences due to a high level of anthropization. However, greater decreases in connectivity are observed in the east (Fig. 3b). Notably, focusing on the potential corridors in the current condition reveals an increase in connectivity in future scenarios, especially near the Partenio Regional Park (P-RP; Campania region) (Fig. 3b).

## Discussion

In this study, using habitat and connectivity modeling for the genus *Salamandrina*, we identified the areas where the two species*, S. terdigitata* and *S. perspicillata*, may face future extinction and those where they are likely to persist. Additionally, we pinpointed potential ecological corridors that could enhance connectivity among populations. Habitat suitability projections for both species indicate a consistent northwestward shift. However, *S. perspicillata* is expected to experience reduced habitat connectivity, whereas *S. terdigitata* may see increased connectivity.

Amphibians are declining at an alarming rate, with 43% of the species currently listed as threatened (IUCN Red List: Critically Endangered, Endangered, Vulnerable), up from 37.9% in 1980 and 39.4% in 2004. Climate change, responsible for 39% of status deterioration since 2004, and habitat loss (37%) are key drivers [5], and among the main threats to the genus *Salamandrina*, as well as the spread of diseases, invasive alien species, and aquatic pollution [5, 16]. Thus, finding the most important areas where a species could persist in the future is one measure that could be implemented, and species distribution models have already been used to assess crucial conservation areas for several species [52–54].

Our predictions align with those obtained by previous studies [8, 9], showing, in general, several suitable patches persisting along the Apennines. On a finer scale, our findings suggest a general preference for moist habitats, like humid forests and valleys, consistent with the species’ ecology [43, 44, 55]. The weighted suitability maps we generated could serve as a valuable tool to support further research on the distribution of both species. Due to their life cycle and a more marked terrestrial activity phase only during certain months of the year [6, 43, 56], these species are challenging to observe. Therefore, our maps can provide new insights into the distribution of these endemic species, especially in light of recent evidence showing new records at the northern limit of the range for *S. perspicillata* [57].

Future projections indicate a similar pattern of range shifts northwestward. These directional and long-range shifts align with patterns reported in other ecological modeling studies, where a prevailing northwestern displacement is observed in the Saharan-Arabic region, and a northeastern shift occurs in the Palaearctic [58–60]. These projected changes are consistent with the ectothermic nature of amphibians, a physiological trait which makes them particularly susceptible to the impacts of climate change [61]. Despite their sensitivity to climate change, both species may adapt to some extent, as evidenced by studies on other Salamandridae showing morphological adaptations to changing climates [62, 63].

Our models predict that *S. terdigitata* will have its main stable areas in three main distinct zones: between Campania and Calabria, in the Sibari plain (northern Calabria, at the foot of Pollino Massif) and at the tip of Calabria (at the foot of the Aspromonte Massif). These areas largely overlap with those identified in previous modeling studies [8, 9]. For *S. perspicillata*, we identified a critical area between Campania and southern Latium in the Volsci Mountains. Interestingly, this is the same area that could have acted as glacial refugia [7], underlining its importance for the conservation of this species.

The post-glacial expansion and the secondary contact inferred through genetic data [7, 15] are also supported by our future projections (Fig. 1b). Our ecological corridors predict the northward expansion of *S. perspicillata* through the Apennines, which aligns well with routes used for the recolonization of the Northern Apennines [9]. Similarly, the increased connectivity observed in southern Latium corresponds to a previous study modeling the past distribution of the genus *Salamandrina* [9]. This further confirms the importance of this area, not only for past colonization dynamics but also for the future. For *S. terdigitata*, we found greater connectivity in the northern part of its range (Fig. 2a), similarly to previous studies that showed low use of corridors in the southern part of *S. terdigitata’*s range, such as the Calabrian Apennines [9]. Furthermore, this aligns with the genetic data of Hauswaldt et al. [13], who estimated no contacts among populations during the post-glacial period. However, since the ‘pairwise’ mode in Circuitscape was applied to compute the connectivity models, the consequent results can be influenced by the focal nodes used, and this point should be considered when inspecting our map, for management and conservation actions. Furthermore, our connectivity models are based on a resistance layer that reflects the assumption that salamanders disperse and move preferably along high-quality habitats, which, in certain cases, can be a poor predictor of gene flow [64].

It is hypothesized that *S. perspicillata* recolonized the Central Apennines from southern Latium, while *S. terdigitata* expanded into the Campanian Apennines likely facilitated by improved climatic conditions during the Mid-Holocene period [9]. This range expansion led to the formation of a sympatric zone, as hypothesized by Mattoccia et al. [7] and Canestrelli et al. [15], enabling a “secondary contact” between the two species, under post-glacial conditions. Previous genetic studies have demonstrated that introgressive hybridization occurred in this region in the past [7, 14, 15]. Our connectivity models within this contact area suggest that this process may persist or even intensify in the future. Specifically, our projections indicate a northward shift in habitat suitability for *S. terdigitata*, alongside the presence of potential ecological corridors that may facilitate further contact with *S. perspicillata*. Therefore, the importance of future monitoring efforts should be focused on this area, to assess whether hybridization is ongoing and potentially increasing. From this perspective, our models provide a valuable tool for identifying priority areas where conservation actions should be prioritized.

Since for future predictions we considered only climate-change scenarios (i.e., changing the bioclimatic predictors and keeping fixed the others), our models can potentially result over-optimistic. Indeed, one should consider the current lack of fine-scale future projections for habitat changes and river flow dynamics, as the available ones [e.g., 65] are restricted to specific future scenarios and their spatial resolution does not meet the one needed to infer small-scale events and, possibly, management needs. Furthermore, predicting changes in variables such as river discharge and forest cover remains particularly challenging to predict, as they may respond to climate change with a considerable time-lags and high spatio-temporal variability [e.g., 66]. Nevertheless, we highlight the value of incorporating such non-bioclimatic predictors in future studies to refine ecological modeling at finer spatial scales, to enhance the realism and applicability of fine-scale ecological models beyond purely climate-driven changes. This could help evaluate, for instance, the role of microhabitat characteristics and local microclimatic conditions. Further research could focus on these localized factors to better understand how they influence the persistence and distribution of the target species. The low extinction rates of Pleistocene amphibians (when compared to those of contemporary birds and mammals) could indirectly suggest that the long-term survival of amphibian species could depend on even small populations with limited ranges [67], as in the case of the populations of *Salamandrina* surviving in refugia of central and southern Italy [9]. Therefore, in the case of these species, local-scale conservation efforts might successfully ensure their long-term survival. Considering the future perspectives for these small inhabitants of mesophilous or subthermophilous woods, efforts to mitigate future global warming remain critically important. However, given the aforementioned resilient nature of amphibian species [68], local governments should prioritise forest and river management plans to favor habitat suitability for these salamanders, such as those suggested by Basile et al. [43]. These plans include maintaining a suitable number of trees with a trunk diameter exceeding 30 cm, implementing selective logging along the banks of streams and leaving an adequate buffer zone around streams [43, 56]. Conservation efforts preserving habitat suitability in the last refugia of these species (i.e., Southern Latium and Calabria) are crucial due to their biological uniqueness, especially when some decreases are observed from long-term field assessments as for a 40-year assessment for *S. terdigitata* in Calabria [69]. These taxa are strikingly different from other salamanders, for example, in their vestigial lungs. Also, they exhibit a suite of unique behavioral traits, making them the only urodeles able to perform the unkenreflex (i.e., curling the body dorsally to reveal their ventral color pattern as a defensive behavior [70]), to display the so-called “stand up behavior” by males, and for their peculiar courtship behavior [71]. These peculiarities contribute to a high degree of uniqueness and ‘distinctiveness’ of this taxon, significantly enhancing the taxonomic diversity and the morphological disparity of the ecosystems that include them [72]. Therefore, from a conservation biology perspective, a responsible and far-sighted wildlife management plan should prioritize the irreplaceable areas where these species occur, even if the region is not considered vulnerable in the short term [73].

Similarly, identifying and maintaining ecological corridors could be crucial for supporting the long-term survival of these species. This study represents a first step by assessing habitat suitability and connectivity at a fine resolution. Future research should evaluate whether critical habitats are adequately covered within the existing protected area network. If gaps are identified, establishing new protected areas may be necessary to align with the Kunming–Montreal Global Biodiversity Framework’s 30 × 30 target.

## Supplementary Information

Additional file 1.

## Data Availability

The data underlying this article will be shared on reasonable request to the corresponding author.
