# Supplementary material for: Modeling habitat suitability and connectivity for the sole endemic genus of Italian vertebrate: present and future perspectives
Source: Front Zool. 2025 May 26;22:8. doi: 10.1186/s12983-025-00562-6 (PMC12105252; doi:10.1186/s12983-025-00562-6)
Supplement: Supplementary file 1 — Additional file 1. [file 12983_2025_562_MOESM1_ESM.docx]

**Supplementary Information of the article:**

**“Modeling habitat suitability and connectivity for the sole endemic genus of Italian vertebrate: present and future perspectives”**


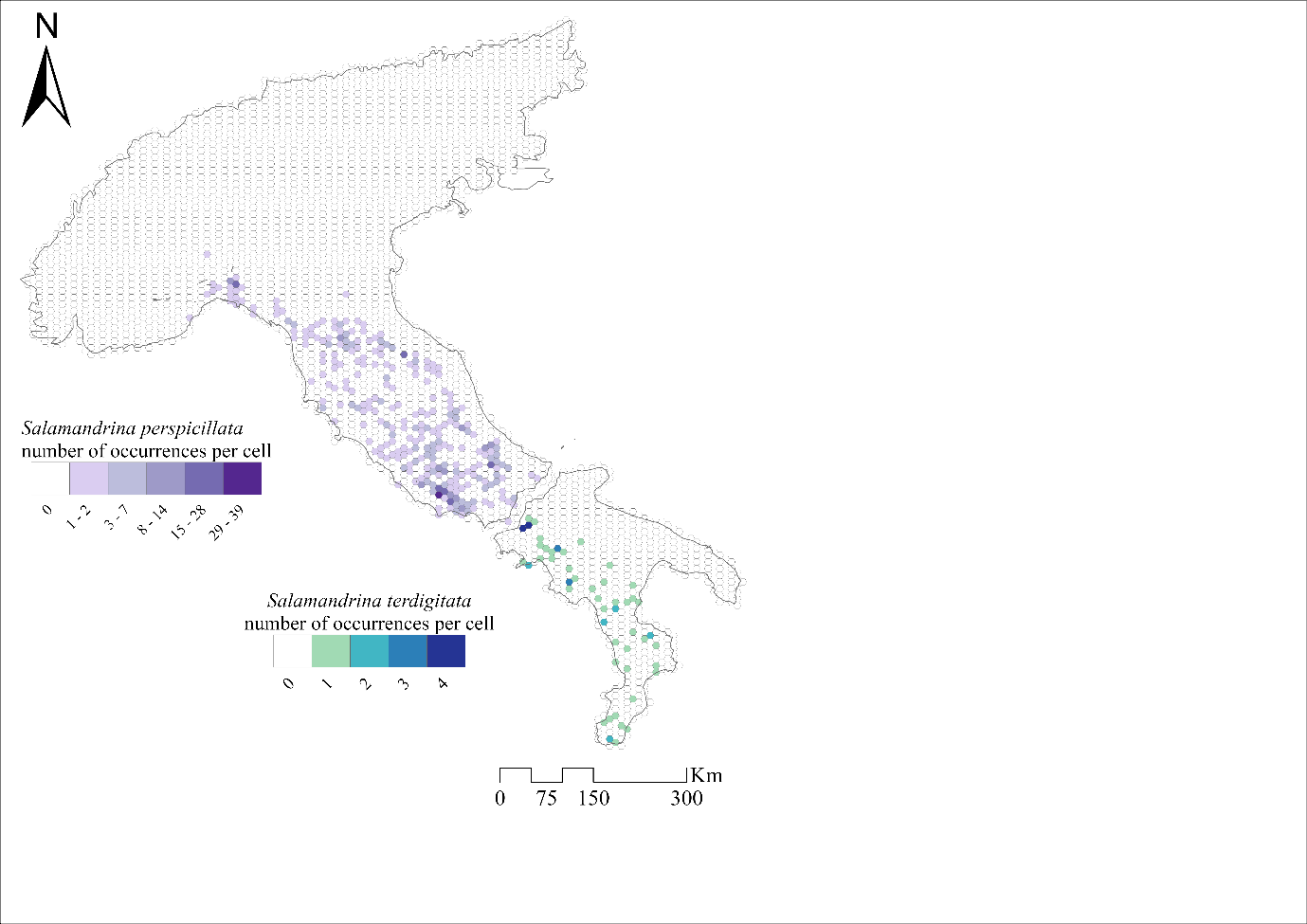


**Figure S1.** Number of occurrences for S*alamandrina perspicillata* and *S. terdigitata* used to calibrate the species distribution models (SDMs).

**Table S1.** Details about the predictor used for the ecological niche modeling (i.e., for the ensemble modeling in ‘biomod2’) (light grey), and during the fine-scale modeling in ArcGIS Pro (grey).

| **Predictor** | **Source** | **Details** |
| --- | --- | --- |
| WorldClim bioclimatic variables | <https://www.worldclim.org/data/bioclim.html> | Used at their original resolution for the ecological niche modeling procedure within ‘biomod2’. At the latitude of the study area, the resolution is ~700m (i.e., ~1km at the equator). |
| Slope | <https://sdi.eea.europa.eu/catalogue> | Starting from the reported Digital Elevation Model (DEM) at 25m resolution, we used the ’Surface Parameters’ tool in ArcGIS Pro to calculate the Slope. The Slope was then resampled at 100m resolution. |
| Aspect | <https://sdi.eea.europa.eu/catalogue> | Starting from the reported Digital Elevation Model (DEM) at 25m resolution, we used the ’Surface Parameters’ tool in ArcGIS Pro to calculate the Aspect. The Aspect was then resampled at 100m resolution. |
| EUNIS Habitat Map | https://www.eea.europa.eu/data-and-maps/data/ecosystem-types-of-europe | Downloaded from the European Environment Agency at 100m resolution. |
| Distance from rivers | https://www.hydrosheds.org/products/hydrorivers | After downloading the dataset, we selected small tributaries and streams, which are more important for the species, filtering them with the Strahler order (>3). Then, we used the ‘Euclidean Distance’ tool within ArcGIS Pro to calculate the Euclidean distance at a resolution of 100m. |
| Distance from pools, springs and drinking-troughs | Dataset compiled by the authors | After compiling the dataset, we used the ‘Euclidean Distance’ tool within ArcGIS Pro to calculate the Euclidean distance at a resolution of 100m. |

**Table S2.** Details about the specific transformations applied to each environmental predictor and the relative weight assigned, within the Suitability Modeler tool in ArcGIS Pro.

| **Predictor** | **Transformation** | **Weight** |
| --- | --- | --- |
| Weighted Ensemble | Linear | 40 |
| EUNIS Habitat Map | Categorical – Class by class | 26 |
| Slope | Logistic Growth | 12 |
| Aspect | Inverse Logistic Growth | 12 |
| Distance from rivers | Linear | 5 |
| Distance from pools, springs and drinking-troughs | Linear | 5 |

**Figure S2.** Correlograms showing, for each species (*S. perspicillata*, *S. terdigitata*) × algorithm (GLM, GBM, RF) combination, the variation, at increasing inter-point distance, of Moran’s index (Moran’s I) computed on the residuals of the preliminary models fitted for each presence-pseudoabsence dataset. The dashed horizontal red lines (-0.1 < Moran’s I < 0.1) help in identifying inter-point distance where Moran’s I approach 0 (i.e., the spatial autocorrelation range).
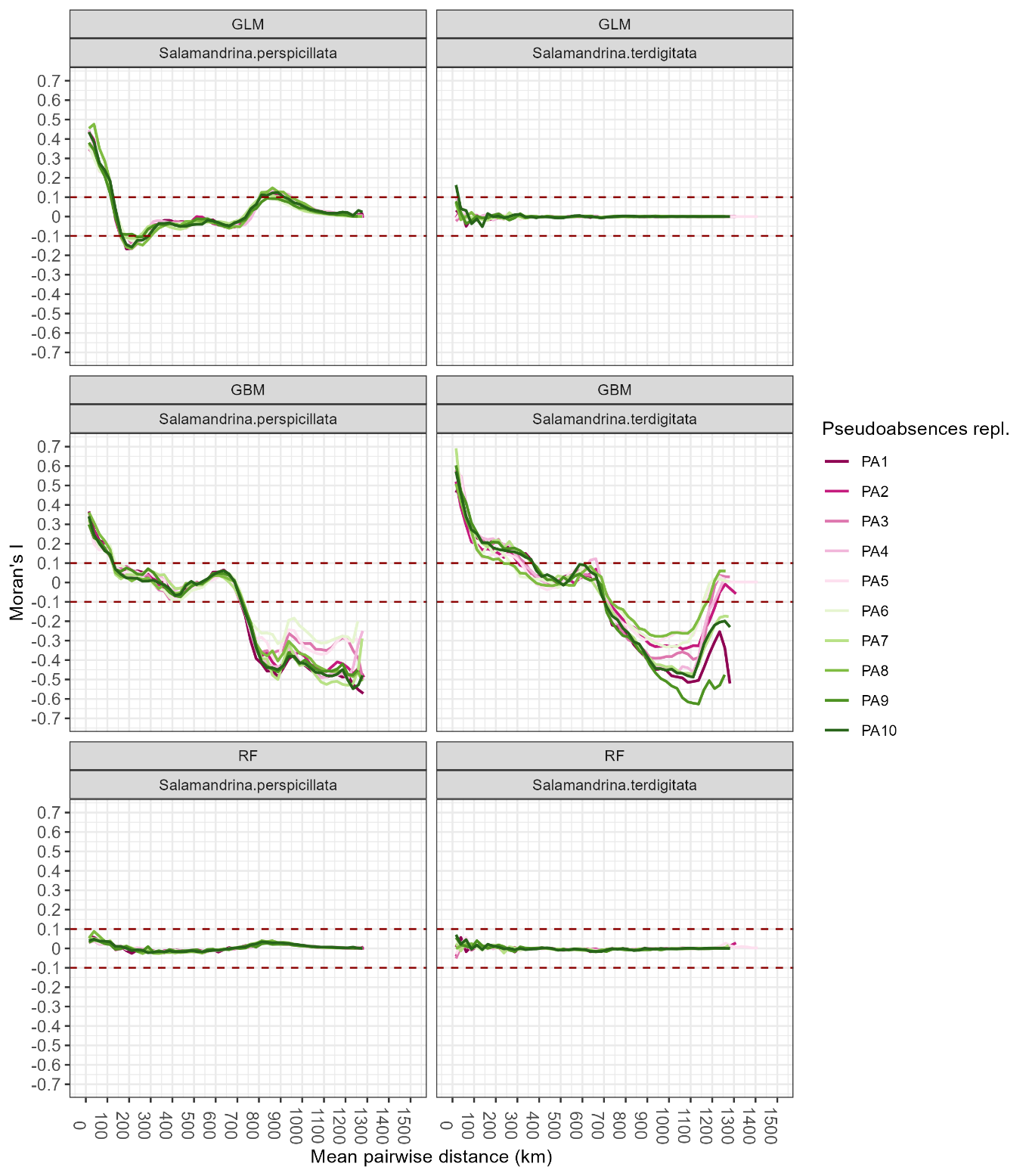


**Figure S3.** Checkerboard blocking structure implemented for *S. perspicillata* (left) and *S. terdigitata* (right). Block size (= 400 km) was chosen based on the spatial autocorrelation range estimated through the correlograms derived on residuals from preliminary SDMs fitted for both species. Black triangles show occurrence points considered for SDMs’ fitting and validation.
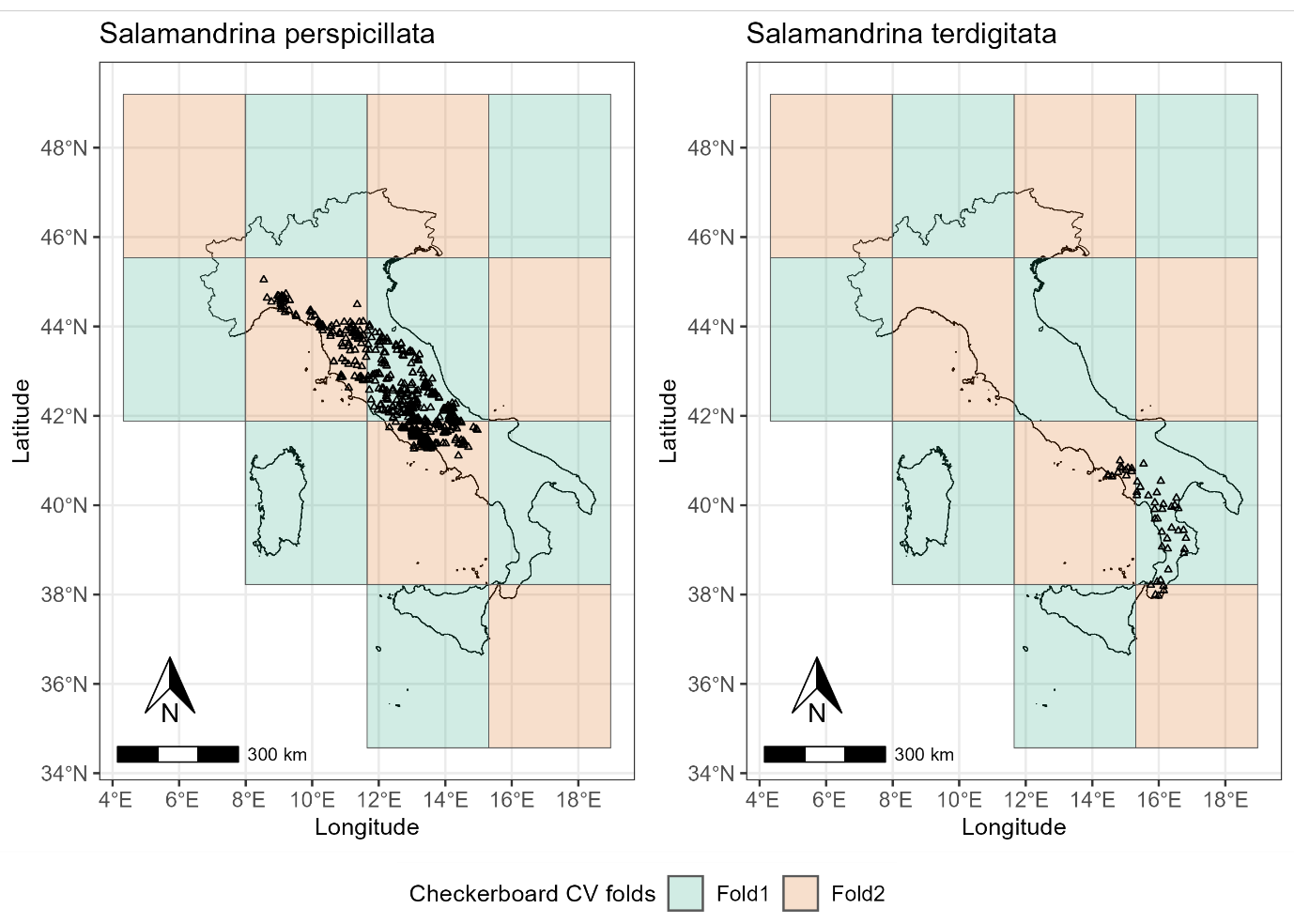


**Figure S4.** Climatic suitability across the study area for *S. perspicillata* (left) and *S. terdigitata* (right), according to the weighted mean ensemble projection of the SDMs attaining Boyce index >= 0.7 on checkerboard CV test data. Red triangles show occurrence points considered for SDMs’ fitting and validation.


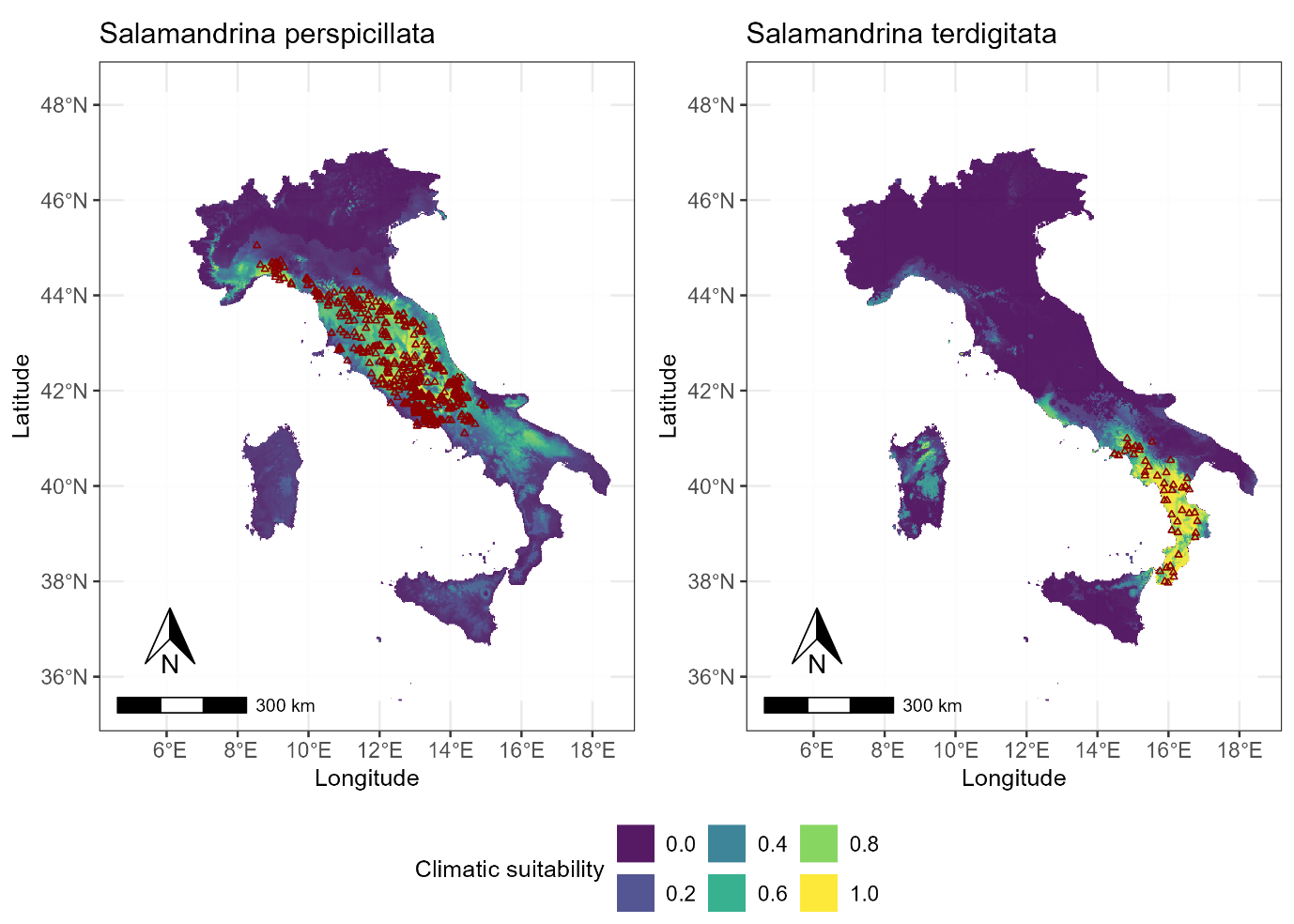


**Figure S5.** Marginal response curves (smoothed by applying a GAM algorithm to predicted values: blue line represents the mean value while the grey ribbon shows the confidence interval) for two variables (one related to temperature and the other to precipitation) out of the three variables with the highest relative importance scores within the Boyce-weighted ensemble model obtained for *S. perspicillata* (top row) and *S. terdigitata* (bottom row). bio10 = mean temperature of warmest quarter; bio17= precipitation of the driest quarter; bio19: precipitation of the coldest quarter.


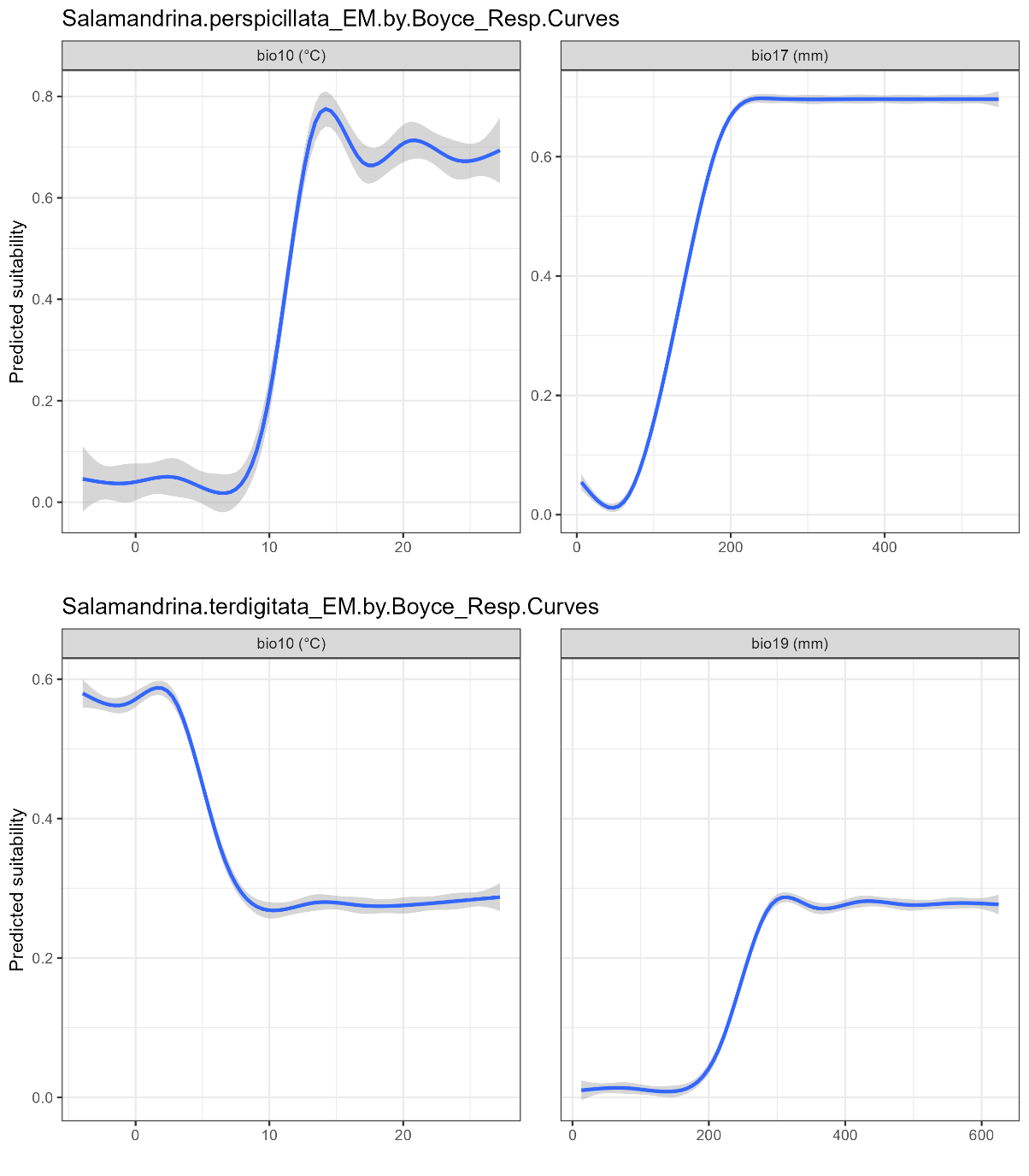


**Figure S6.** Predicted ranges’ shifts of weighted suitability obtained by comparing the models from the current conditions and each of the future scenarios, for *S. perspicillata*.

**
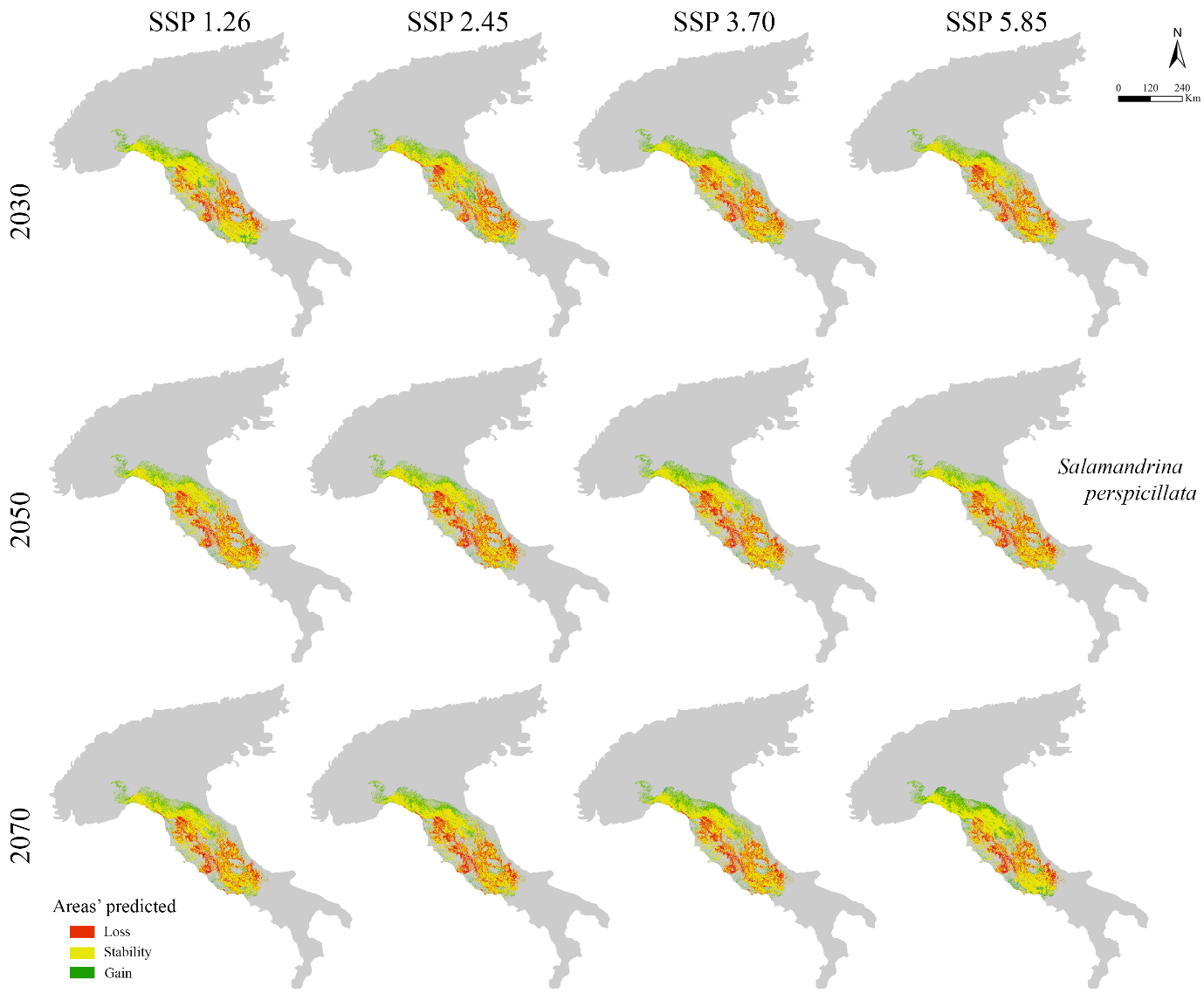
**

**Figure S7.** Predicted ranges’ shifts of weighted suitability obtained by comparing the models from the current conditions and each of the future scenarios, for *S. terdigitata*.

**
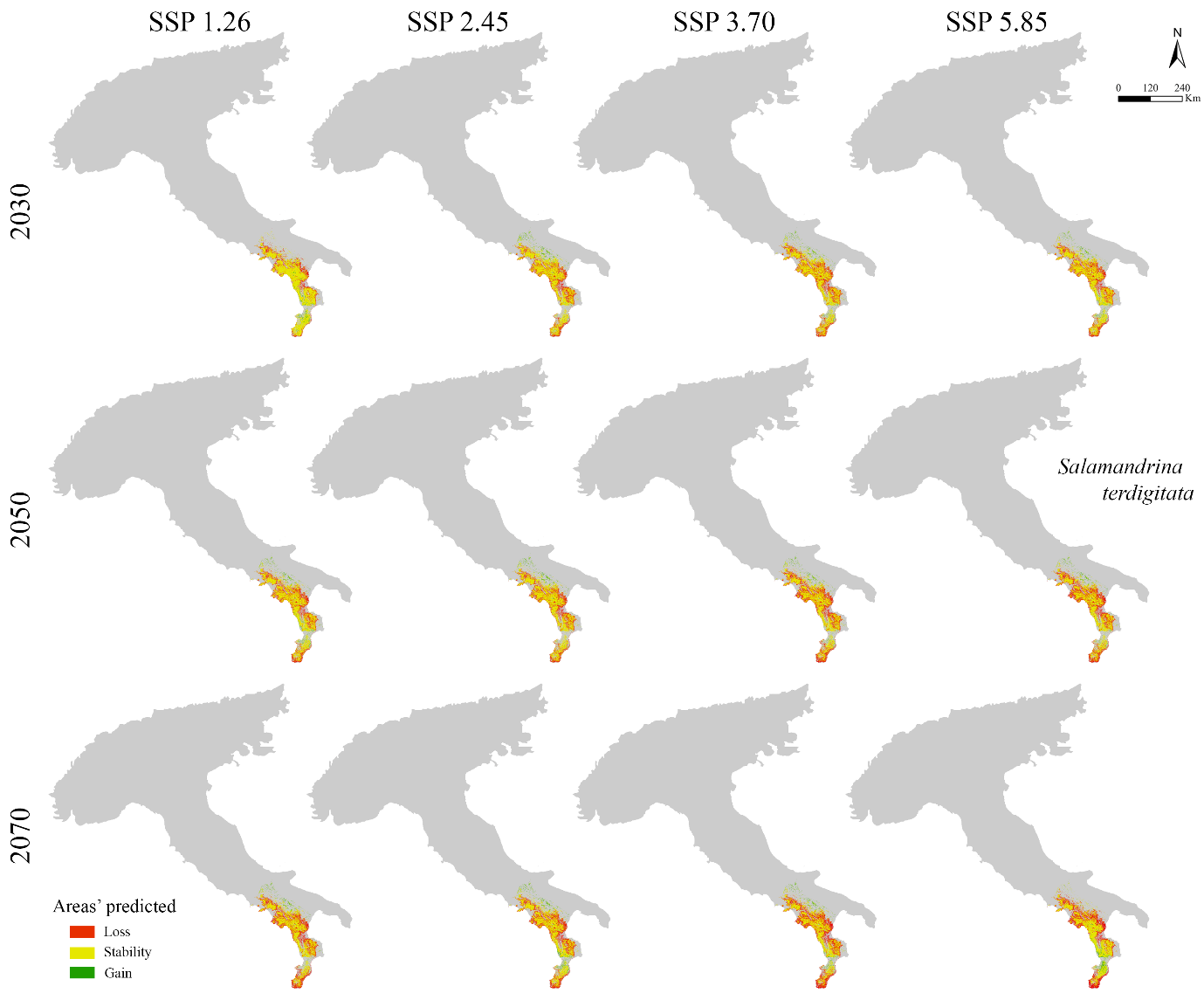
**
